# Effectiveness of inactivated SARS-CoV-2 vaccine (CoronaVac) on intensive care unit survival

**DOI:** 10.1017/S0950268822000267

**Published:** 2022-02-09

**Authors:** Sultan Acar Sevinc, Seyhan Metin, Nermin Balta Basi, Jonathan Ling, Ayse Surhan Cinar, Sibel Oba

**Affiliations:** 1 Anesthesiology and Reanimation Department, Sisli Hamidiye Etfal Education and Training Hospital, Istanbul, Turkey; 2 Faculty of Health Sciences and Wellbeing, University of Sunderland, Sunderland, UK

**Keywords:** CoronaVac, COVID-19, SARS-CoV-2 pandemic, Sinovac, vaccination

## Abstract

This study compared the course of coronavirus disease 2019 (COVID-19) in vaccinated and unvaccinated patients admitted to an intensive care unit (ICU) and evaluated the effect of vaccination with CoronaVac on admission to ICU. Patients admitted to ICU due to COVID-19 between 1 April 2021 and 15 May 2021 were enrolled to the study. Clinical, laboratory, radiological parameters, hospital and ICU mortality were compared between vaccinated patients and eligible but unvaccinated patients. Patients over 65 years old were the target population of the study due to the national vaccination schedule. Data from 90 patients were evaluated. Of these, 36 (40.0%) were vaccinated. All patients had the CoronaVac vaccine. Lactate dehydrogenase and ferritin levels were higher in an unvaccinated group than vaccinated group (*P* = 0.021 and 0.008, respectively). SpO_2_ from the first arterial blood gas at ICU was 83.71 ± 19.50% in vaccinated, 92.36 ± 6.59% in unvaccinated patients (*P* = 0.003). Length of ICU and hospital stay were not different (*P* = 0.204, 0.092, respectively). ICU and hospital mortality were similar between groups (*P* = 0.11 and 0.70, respectively). CoronaVac vaccine had no effect on survival from COVID-19. CoronaVac's protective effect, especially on new genetic variants, should be investigated further.

## Introduction

Coronavirus disease 2019 (COVID-19) is caused by severe acute respiratory syndrome coronavirus 2 (SARS-CoV-2) infection. It has caused more than 5 million deaths worldwide and 73 000 deaths in Turkey [1]. There is no specific therapy for COVID-19 so vaccines are the major hope for the control of the disease.

According to World Health Organization data, there are 287 candidate vaccines for COVID-19 [2]. Twenty-one types of vaccines have been used for prevention to date [1]. Turkey started to vaccinate healthcare professionals in January 2021 with the CoronaVac vaccine and declared a step by step inclusion schedule for the national vaccination programme [3, 4].

CoronaVac is an inactivated viral vaccine which has been found effective in Phase III trials [5, 6]. However, few studies have investigated the effect of CoronaVac on the incidence and clinical course of the disease.

The aim of this study is to compare clinical, laboratory and survival data of COVID-19 patients who had been vaccinated at least 14 days before enrolment to the study with COVID-19 patients who had not been vaccinated although they were eligible.

## Methods

### Design and participants

This is a retrospective cross-sectional study performed in accordance with the ethical standards of the Declaration of Helsinki. Local Ethical Committee approval number is 1903 granted 25 May 2021 (NCT04956562). Informed consent was taken from patients or legally acceptable representatives for the use of their medical data.

Patients admitted to our institution's intensive care unit (ICU) according to the national guideline on behalf of the Ministry of Health between 1 April 2021 and 15 May 2021 was enrolled to the study [7]. Exclusion criteria were admissions to ICU due to reasons other than COVID-19, age younger than 18, patients with negative real-time polymerase chain reaction (RT-PCR) result for SARS-CoV-2, continued admission at the end of enrolment date, admission of healthcare professionals due to COVID-19.

At the start of the national vaccination programme, CoronaVac was the only available type of vaccine and given at 2 doses 28 days apart. The injection was 0.5 ml containing 600 SU of SARS-CoV-2 antigen [8].

The time for seroconversion was accepted at least 2 weeks after the second dose of the vaccine. This means that, to be eligible, a patient must have their first dose of vaccine before 18 February 2021. After healthcare professionals, Turkey has started to vaccinate citizens over 90 years old and decreased the age cut-off 5 years at every step with irregular intervals. Vaccination of citizens over 65 years started on 12 February 2021 and citizens over 60 years on 28 March 2021. These details mean that enrolled patients would be at least 65 years old. As a result, vaccinated COVID-19 patients over 65 years comprised the study group; unvaccinated patients in the same age group were the control group. Patients younger than 65 years of age were naturally excluded from the study because the national programme had not let them be vaccinated.

Vaccination was accepted as complete if the patients had two doses. The antibody response was expected to be enough 2 weeks after the second dose. Patients without any vaccination or with only one dose of vaccine were accepted as unvaccinated. Analyses were also performed when patients with one dose vaccine were excluded from the study population.

### Data collection

For the primary research question, patients' age and sex, history of co-morbidities including diabetes mellitus, hypertension, coronary artery disease, chronic pulmonary obstructive disease vaccination dates and types were noted from the hospital record system. Laboratory parameters collected for evaluation at admission to ICU were urea, creatinine, haemoglobin, haematocrit, leucocyte, lymphocyte, neutrophil, thrombocyte, C-reactive protein, procalcitonin, ferritin, D-dimer, alanine aminotransferase, lactate dehydrogenase, and details of first arterial blood gas at ICU (pH, pO_2_, pCO_2,_ spO_2_), *P*_aO2_/*F*_IO2_ ratio. Glasgow Coma Score was calculated in all patients except ones already intubated at admission. Systolic and diastolic blood pressure were also noted. The initial respiratory condition was classified as room air, nasal oxygen support, non-invasive mechanical ventilation (NIMV) and invasive mechanical ventilation (IMV). The frequency of patients intubated at follow-up was calculated. Days to intubation, duration of intubation, length of ICU and hospital stay were noted.

The last status of patients at discharge from ICU and hospital were used to compare ICU and hospital mortality. ICU and hospital mortality analysis was repeated as if the time for seroconversion was accepted as 28 days instead of 14 days. Therefore, the analysis was performed for patients admitted to ICU due to COVID-19 between 15 April and 15 May 2021.

The data that support the findings of this study are openly available in Zenodo at https://doi.org/10.5281/zenodo.5730446 [9].

### Statistical analysis

Statistical analyses were performed with the Scientific Package for Social Science (version 21.0; SPSS Inc., Chicago, IL, USA). Continuous variables were given as mean ± standard deviation if they distributed normally or as median (interquartile range) if they were distributed abnormally. Qualitative variables were given as a percentage. Comparison of normally distributed data was performed by independent samples *t*-test. Abnormally distributed data were compared with the Mann–Whitney *U* test. Categorical variables were compared by the χ^2^ test. Differences were considered statistically significant for *P* values less than 0.05. Survival analysis was performed by the Kaplan–Meier curve.

## Results

**Fig. 1.**
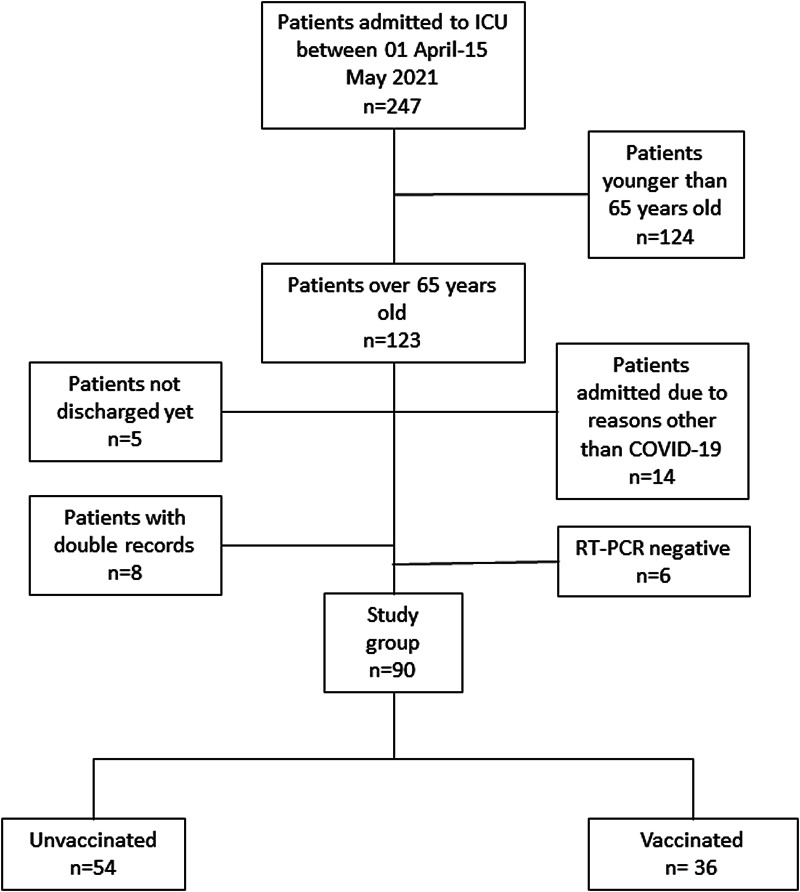
Details of the patient cohort. Patients admitted to the intensive care unit from 1 April- 15 May 2021 were included to the study.

In total, 247 patients were admitted to the ICU of our institution between 1 April and 15 May 2021. Details of the enrolment process are given in Figure 1. No healthcare professionals were admitted to ICU with COVID-19 during the enrolment period. Data from 90 patients met the inclusion criteria and were evaluated. Vaccinated patients were 40.0% (*n* = 36) of the study population. The unvaccinated patient group (60.0%, *n* = 54) consisted of patients with one dose of vaccine (31.48%) and no vaccine at all (68.51%). All patients had been given the CoronaVac vaccine.

Demographic characteristics of patients are given in Table 1. Age, sex, co-morbidities, blood pressure and Glasgow Coma Scale at admission to ICU were similar between vaccinated and unvaccinated patients. Laboratory values, except ferritin and lactate dehydrogenase, were similar between groups (Table 2). Ferritin level was 469.50 (229.52–884.10) ng/ml in vaccinated and 816.50 (368.45–1340) ng/ml in unvaccinated patients (*P* = 0.008). Lactate dehydrogenase levels were 340 (252–538) U/l and 461 (360–609) U/l in vaccinated and unvaccinated patients, respectively (*P* = 0.021). Arterial oxygen saturation (spO_2_) at admission to ICU was higher in unvaccinated (92.36 ± 6.59%) than in vaccinated patients (83.71 ± 19.50%) (*P* = 0.003) (Table 3). Respiratory support types were similar between groups (*P* = 0.135). Intubation rates at admission and during clinical follow-up for vaccinated and unvaccinated patients were similar (*P* = 0.60, 0.055). Overall intubation frequency was 72.20% in vaccinated and 85.20% in unvaccinated patients (*P* = 0.132). *P*_aO2_/*F*_IO2_ ratio at admission for vaccinated and unvaccinated patients were 80 (40–105) %, 90 (60–150) %, respectively (*P* = 0.071). Duration of ICU and hospital stay were similar between groups (*P* = 0.204, 0.092, respectively).

**Table 1.** Demographic and clinical characteristics of patients

	All patients, *n* = 90	Vaccinated, *n* = 36	Unvaccinated, *n* = 54	*P*
Age, years, mean ± s.d.	79.23 ± 8.66	81.08 ± 7.49	78 ± 9.22	0.098
Female, *n* (%)	48 (53.3)	15 (41.7)	33 (61.1)	0.07
Co-morbidities, *n* (%)				
Hypertension	72 (80)	29 (80.6)	43 (79.6)	0.914
DM	38 (42.2)	18 (50)	20 (37)	0.223
CAD	49 (54.4)	20 (55.6)	29 (53.7)	0.863
COPD	18 (20)	7 (19.4)	11 (20.4)	0.914
Blood pressure, mmHg, mean ± s.d.				
Systolic	120.10 ± 29.13	118.50 ± 31.97	121.17 ± 27.33	0.673
Diastolic	65.39 ± 13.97	64.58 ± 14.90	65.93 ± 13.43	0.658
GCS, median (IQR)	15 (12–15)	15 (12–15)	15 (12–15)	0.712

CAD, coronary artery disease; COPD, chronic obstructive pulmonary disease; DM, diabetes mellitus; GCS, Glasgow coma scale; IQR, interquartile range; s.d., standard deviation.

**Table 2.** Laboratory parameters of patients at admission to an intensive care unit

Parameter	All patients, *n* = 90	Vaccinated, *n* = 36	Unvaccinated, *n* = 54	*P*
Leucocyte, × 10^9^/l, median (IQR)	11.00 (7.84–14.68)	10.71 (7.57–13.7)	11.28 (7.84–15.32)	0.592
Neutrophile, × 10^9^/l, median (IQR)	9.77 (6.72–12.93)	9.61 (5.94–12.55)	9.77 (6.85–13.04)	0.374
Lymphocyte, × 10^9^/l, median (IQR)	0.58 (0.32–1.04)	0.64 (0.39–1.46)	0.52 (0.30–0.81)	0.122
Haemoglobin, g/dl, median (IQR)	11.35 (10.10–13.02)	11.15(9.82–12.70)	11.45 (10.60–13.15)	0.376
Haematocrit, %, mean ± s.d.	35 ± 6.26	34.02 ± 5.87	35.64 ± 6.48	0.231
Thrombocyte, × 10^9^/l, mean ± s.d.	252.02 ± 111.21	244.92 ± 95.24	256.76 ± 121.33	0.623
Urea, mg/dl, median (IQR)	69 (54–118.75)	73.5 (57.5–138.5)	66.5 (45.75–111)	0.236
Creatinine, mg/dl, median (IQR)	1.10 (0.84–2.26)	1.22 (0.92–3.31)	0.99 (0.78–1.67)	0.051
Alanine aminotransferase, U/l, median (IQR)	21.50 (14–34)	20.5 (15–34)	23 (13.75–35)	0.895
Lactate dehydrogenase, U/l, median (IQR)	418 (297.50–579.75)	340 (252–538)	461 (360–609)	**0**.**021**
C reactive protein, mg/l, median (IQR)	116.10 (67.02–190)	114.50 (88.97–188.40)	117.35 (66.18–191.61)	0.714
Procalcitonin, ng/ml, median (IQR)	0.47 (0.15–1.30)	0.50 (0.16–1.10)	0.46 (0.14–1.49)	0.615
Ferritin, ng/ml, median (IQR)	663.5 (347.75–1209)	469.50 (229.52–884.10)	816.50 (368.45–1340)	**0**.**008**
D-dimer, ng/ml, median (IQR)	1120 (560–1992)	1085 (491.75–2538.75)	1170 (599.25–1792.25)	0.754

IQR, interquartile range; NIMV, non-invasive mechanical ventilation; s.d., standard deviation.

**Table 3.** Arterial blood gas analysis, details of respiratory function at admission and follow-up, length of stay at ICU and hospital for all patients and subgroups

	All patients, *n* = 90	Vaccinated, *n* = 36	Unvaccinated, *n* = 54	*P*
Arterial blood gas				
pH, mean ± s.d.	7.37 ± 0.12	7.38 ± 0.10	7.36 ± 0.13	0.483
PaCO_2_, mmHg, median (IQR)	36.60 (30.32–42.62)	35.8 (29.87–44.35)	36.70 (30.55–42.15)	0.837
PaO_2_, mmHg, median (IQR)	76.70 (57.07–114.05)	70.70 (48.02–120.75)	77.10 (60.40–110.275)	0.313
SpO_2_, %, mean ± s.d.	88.96 ± 13.82	83.71 ± 19.50	92.36 ± 6.59	**0**.**003**
*P*_aO2_/*F*_IO2_ ratio	80 (50–120)	80 (40–105)	90 (60–150)	0.071
Respiratory condition at admission, %				0.135
Room air	1.1	2.8	–	
Nasal oxygen	11.1	2.8	16.7	
NIMV	46.7	50	44.4	
IMV	41.1	44.4	38.9	
IMV, overall, %	80	72.2	85.2	0.132
Time to intubation, days, median (IQR)	2.5 (1–6.75)	4.5 (1–10.5)	2.5 (1–6)	0.711
Duration of intubation, days, median (IQR)	8 (3–14)	7.5 (3–15.5)	8 (2.5–14)	0.986
Hospital stay, days, median (IQR)	15 (8–24)	11 (6.25–22.5)	15 (10–25)	0.092
ICU stay days, median (IQR)	9 (5–14)	7.5 (3–12.75)	10 (6–14)	0.204
ICU mortality rate, %	70	69.4	83.3	0.11
Hospital mortality rate, %	66	75	72.2	0.70

IMV, invasive mechanical ventilation; IQR, interquartile range; NIMV, non-invasive mechanical ventilation; s.d., standard deviation.

All analyses were repeated after excluding patients with one dose of CoronaVac vaccine (Supplementary Table S1). Results were almost always similar to Table 1. The need for IMV during follow-up was 50% in vaccinated, 81% in unvaccinated patients (*P* = 0.037). Comparison of parameters and survival analysis of two groups excluding patients with one dose vaccine was given at Supplementary Table S1 and Supplementary Figure S1, respectively.

**Fig. 2.**
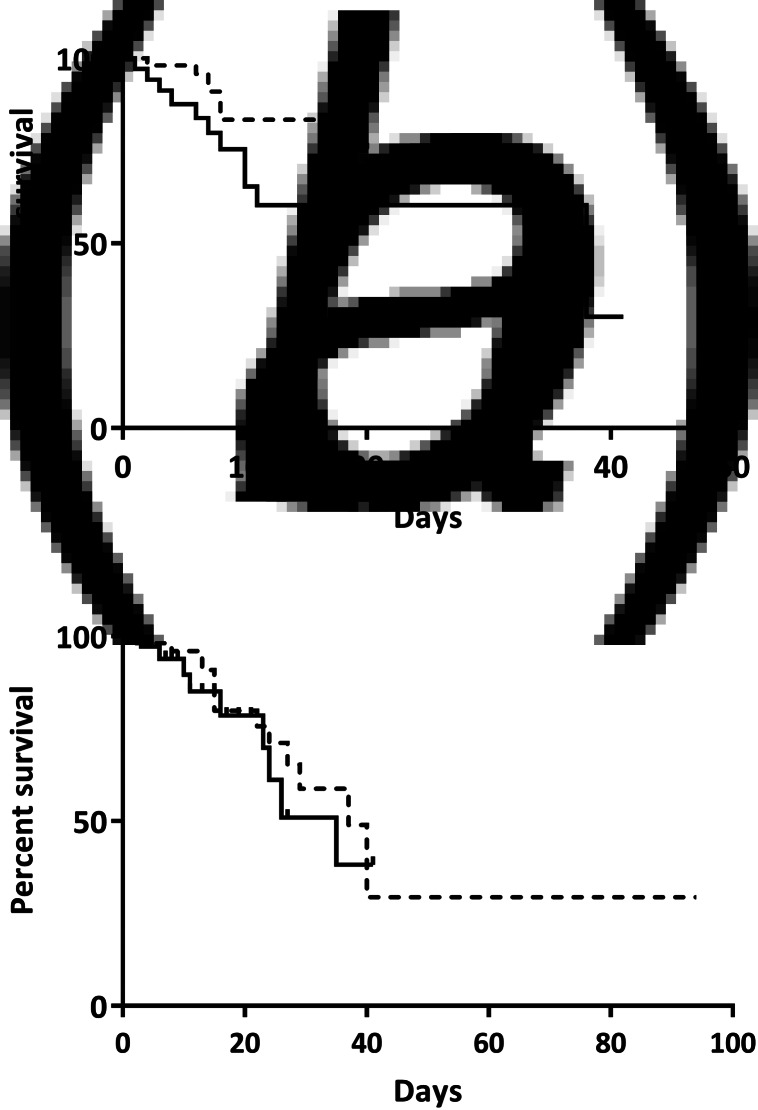
Intensive care unit (a) and hospital mortality (b) if the time for seroconversion is accepted as 14 days. Solid line represents vaccinated, dotted line indicates unvaccinated patients. Mortality between groups was similar at intensive care unit (*P* = 0.11) and hospital (*P* = 0.70).

**Fig. 3.**
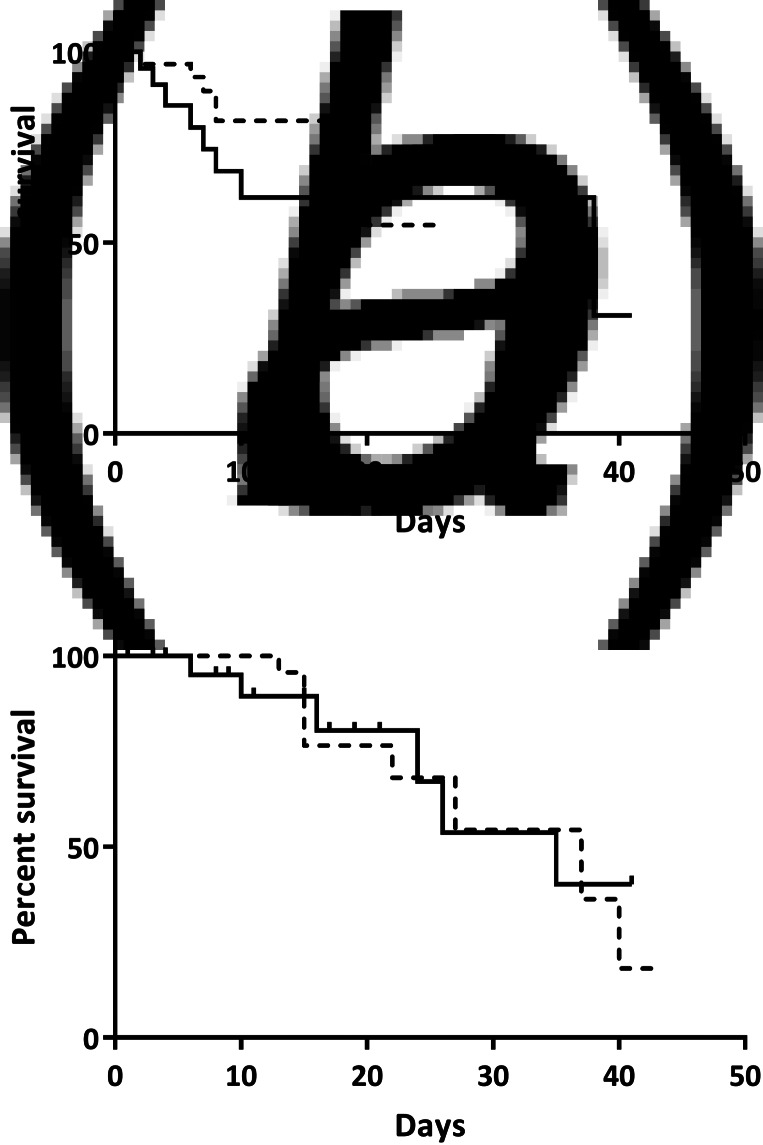
Intensive care unit (a) and hospital mortality (b) if the time for seroconversion is accepted as 28 days. Solid line represents vaccinated, dotted line represents unvaccinated patients. Mortality between groups was similar in intensive care unit (*P* = 0.34) and hospital (*P* = 0.76).

ICU mortality (Fig. 2a) and hospital mortality (Fig. 2b) using 14 days for seroconversion were similar between the two groups (*P* = 0.11, 0.70, respectively). When the time for seroconversion was accepted as 28 days, ICU (Fig. 3a) and hospital mortality (Fig. 3b) between groups also did not differ (*P* = 0.34 and *P* = 0.76, respectively). Comparison of parameters and survival analysis of two groups excluding patients with one dose vaccine is given in Supplementary Table S1 and Supplementary Figure S1, respectively.

## Discussion

We investigated the effectiveness of the CoronaVac vaccine in relation to hospital and ICU mortality and found no differences between vaccinated and unvaccinated groups.

This study gives information about the clinical course of COVID-19 at ICU. We cannot estimate the number of people protected from SARS-CoV-2 infection by CoronaVac vaccine but can conclude that if a patient vaccinated with CoronaVac has been admitted to ICU due to SARS-CoV-2 infection, clinical, laboratory, radiologic parameters were almost always similar to unvaccinated patients. SpO_2_ and *P***_aO2_**/*F***_IO2_** ratio were lower in the vaccinated group indicating that they had worse clinical respiratory conditions than the unvaccinated group. This might be due to several reasons including vaccine effectiveness over 65 years old, effectiveness on different variants, immunogenic response stimulated by a vaccine.

The CoronaVac vaccine was approved by World Health Organization (WHO) and it is the most frequent used vaccine worldwide so far [10, 11]. In Phase III trials, Brazil declared that CoronaVac had prevented hospitalisation and death due to COVID-19 100% starting 14 days after the second vaccination [5]. Turkey declared overall vaccine efficacy against symptomatic SARS-CoV-2 infection as 83.5% compared to 65.3% at Indonesia [6, 10]. Chile has found the effectiveness of the CoronaVac vaccine 90.3% for the prevention of ICU admission [12]. Evidence for the effectiveness of CoronaVac over 65 years is limited. Turkey has excluded patients over 60 years old from phase III trial although they started to vaccinate older patients first. Chile has declared that adjusted vaccine effectiveness for the fully immunised group of individuals aged 60 years and older (two doses, ⩾14 days after the second dose) was 89.2% for ICU admissions [12].

WHO has declared four variants of concern (alpha, beta, gamma, delta) and seven variants of interest [13]. The variants' date of the designation was before enrolment to our study. The effectiveness of the CoronaVac vaccine against SARS-CoV-2 variants is not tested well. Campos *et al*. reported gamma variant in 20 CoronaVac vaccinated patients [14]. Hitchings *et al*. has found adjusted vaccine effectiveness against gamma variant with two-dose CoronaVac vaccination (36.8%) lower than single-dose vaccination (%49.6) [15]. They suspected bias at this study due to inconsistent results. To the best of our knowledge, there are no published data for the effectiveness of a vaccine against delta variant unlike some other vaccines [16–18]. Genomic sequencing of SARS-CoV-2 viruses in Turkey has not been done routinely. However, we know that alpha, beta and gamma variants were present in our country during our study time [19] and 90% of COVID-19 cases were due to gamma variant at August 2021 [20]. Moreover, there is a suspicion that CoronaVac does not protect from the Delta variant because of infected healthcare workers in Indonesia [21], although further evidence is needed to confirm this finding.

CoronaVac provokes an immunogenic response to many viral proteins unlike m-RNA vaccines in which they target spike protein, the protein used to enter cells. Jantarabenjakul has found anti-SARS-CoV-2 total antibody level 185.6 U/ml in CoronaVac, 841.2 U/ml in ChAdOx1 nCoV-19 4 weeks after two-dose vaccinations in patients between 51 and 70 years [22]. Silva *et al*. examined the effectiveness of CoronaVac and ChAdOx1 nCoV-19 vaccines in older people. Vaccine effectiveness at 80–89 years with CoronaVac and ChAdOx1 nCoV-19 was 67.2% and 89.9%, respectively. Above 90 years, it was 65.4% for ChAdOx1 nCoV-19 vaccine and 33.6% for CoronaVac [23]. We do not have antibody titres for our patients, but our patients' antibody titres might be similar to patients without vaccination due to their older age and the nature of the vaccine they had.

Patients with only one dose of CoronaVac were accepted as unvaccinated. Zhang *et al*. found that 28 days after with only one dose of 6 mcg CoronaVac vaccine, seroconversion for neutralising antibody was seen in none and for receptor-binding domain (RBD) specific Ig G in 66.7% of the participants [24]. Seropositivity for S-specific Ig G was detected in 37.5% of participants. Even though the published data favor our decision, all analyses were repeated after the exclusion of patients with one dose vaccine. Analysis results remained the same suggesting there was no selection bias while accepting patients with one dose vaccine as unvaccinated.

Our reference point for antibody formation was at least 14 days like a similar study by Jara *et al*. in which vaccine effectiveness was found 63.4% in patients over 60 years of age [12]. Neutralising antibody formation after vaccination at days 0 and 28 cohort with 6 mcg CoronaVac vaccine was 83.3% at day 14 and 79.2% day 28 after the second dose [7]. In another study, vaccine effectiveness was found as 77.6% 14 days after the second dose of CoronaVac vaccine in 70–74 years of age patients [25]. Survival analysis was repeated taking a time limit to 28 days as well. Both analyses produced similar hospital and ICU mortality. This might mean that the issue about effectiveness and prevention of SARS-CoV-2 infection with the vaccine was not a number of days after the second dose of vaccination.

There was no mortality difference between the two groups. This could be due to reasons mentioned above including ineffectiveness of vaccine at patients over 65 years, insufficient antibody titres and ineffectiveness against some variants. All these suspicions led to third dose vaccination approval with CoronaVac both by WHO and Turkey [10]. This fact makes our findings consistent although we could not sample types of variants or antibody titres in our study.

## Conclusions

If patients vaccinated with CoronaVac were infected with SARS-CoV-2, they had survival similar to unvaccinated patients. We are not alone in finding discouraging results for CoronaVac [26], but further work is required to examine the effects of this vaccine in more depth and with a larger group of participants. Although data were collected from only a single centre and designed cross-sectionally, our study is valuable because it reflects real-world experience related to the use of the CoronaVac vaccine.

## Supplementary material

For supplementary material accompanying this paper visit https://doi.org/10.1017/S0950268822000267. click here to view supplementary material
