# Supplementary material for: Effectiveness of inactivated SARS-CoV-2 vaccine (CoronaVac) on intensive care unit survival
Source: Epidemiol Infect. 2022 Feb 9;150:e35. doi: 10.1017/S0950268822000267 (PMC8886076; doi:10.1017/S0950268822000267)
Supplement: Supplementary file 1 [file hygsup.zip › S0950268822000267sup002.docx]

**Supplementary Table 1. Demographic, laboratory and clinical details of patients without vaccination and two doses of vaccination**

|  | **Vaccinated,**  **n=36** | **Unvaccinated, n=37** | **p** |
| --- | --- | --- | --- |
| **Age, years, mean±SD** | 81.08±7.49 | 79.97±8.47 | 0.555 |
| **Female (%)** | 41.7 | 59.5 | 0.128 |
| **Co-morbidities (%)**  Hypertension  DM  CAD  COPD | 80.6  50  55.6  19.4 | 81.1  37.8  56.8  27 | 0.955  0.295  0.918  0.443 |
| **Blood pressure, mmHg, mean±SD**  Systolic  Diastolic | 118.50±31.97  64.58±14.90 | 120.68±30.21  64.65±14.69 | 0.766  0.065 |
| **GCS, median (IQR)** | 15 (12-15) | 14.5 (11-15) | 0.290 |
| **Leukocyte, x10^9^/L, median (IQR)** | 10.71 (7.57-13.7) | 12.26 (8.31-15.71) | 0.284 |
| **Neutrophile, x10^9^/L, median (IQR)** | 9.61 (5.94-12.55) | 10.93 (7.52-14.73) | 0.171 |
| **Lymphocyte, x10^9^/L, median (IQR)** | 0.64 (0.39-1.46) | 0.51 (0.30-1.03) | 0.212 |
| **Hemoglobin, g/dl, median (IQR)** | 11.15(9.82-12.70) | 11.50 (10.55-13.35) | 0.424 |
| **Hematocrit, %, mean±SD** | 34.02±5.87 | 35.90±6.80 | 0.211 |
| **Thrombocyte, x10^9^/L, mean±SD** | 244.92±95.24 | 260.68±135.31 | 0.568 |
| **Urea, mg/dl, median (IQR)** | 73.5 (57.5-138.5) | 74 (60-117.50) | 0.732 |
| **Creatinine, mg/dl, median (IQR)** | 1.22 (0.92-3.31) | 1.20 (0.89-1.71) | 0.326 |
| **Alanine aminotransferase, U/L, median (IQR)** | 20.5 (15-34) | 19 (13-34.50) | 0.821 |
| **Lactate dehydrogenase, U/L, median (IQR)** | 340 (252-538) | 462 (363-652) | **0.021** |
| **C reactive protein, mg/L, median (IQR)** | 114.50 (88.97-188.40) | 110.00 (66.78-171.00) | 0.519 |
| **Procalcitonin, ng/ml, median (IQR)** | 0.50 (0.16-1.10) | 0.46 (0.14-1.29) | 0.699 |
| **Ferritin, ng/ml, median (IQR)** | 469.50 (229.52-884.10) | 812 (399-1312) | **0.023** |
| **D-dimer, ng/ml, median (IQR)** | 1085 (491.75-2538.75) | 1220 (619-1605) | 0.787 |
| **Arterial blood gas** |  |  |  |
| pH, mean±SD | 7.38±0.10 | 7.35±0.14 | 0.322 |
| PaCO_2_, mmHg, median (IQR) | 35.8 (29.87-44.35) | 36.40 (29.90-42.95) | 0.873 |
| PaO_2_, mmHg, median (IQR) | 70.70 (48.02-120.75) | 77 (62.20-110.85) | 0.283 |
| SpO_2_, %, mean±SD | 83.71±19.5 | 92.33±7.15 | **0.014** |
| **P_aO2_/F_IO2_ ratio** | 80 (40-105) | 80 (60-150) | 0.112 |
| **Respiratory condition at admission, %**  Room air  Nasal oxygen  NIMV  IMV | 2.8  2.8  50  44.4 | -  13.5  43.2  43.2 | 0.287 |
| **IMV, overall, %** | 72.2 | 89.2 | **0.066** |
| **Time to intubation, days, median (IQR)** | 4.5 (1-10.5) | 2 (1.5-6.0) | 0.721 |
| **Duration of intubation, days, median (IQR)** | 7.5 (3-15.5) | 8 (2-13.50) | 0.888 |
| **Hospital stay, days, median (IQR)** | 11 (6.25-22.5) | 15 (8-23) | 0.325 |
| **ICU stay days, median (IQR)** | 7.5 (3-12.75) | 9 (6-14) | 0.386 |
