# Supplementary figures and images for: Effectiveness of inactivated SARS-CoV-2 vaccine (CoronaVac) on intensive care unit survival
Source: Epidemiol Infect. 2022 Feb 9;150:e35. doi: 10.1017/S0950268822000267 (PMC8886076; doi:10.1017/S0950268822000267)

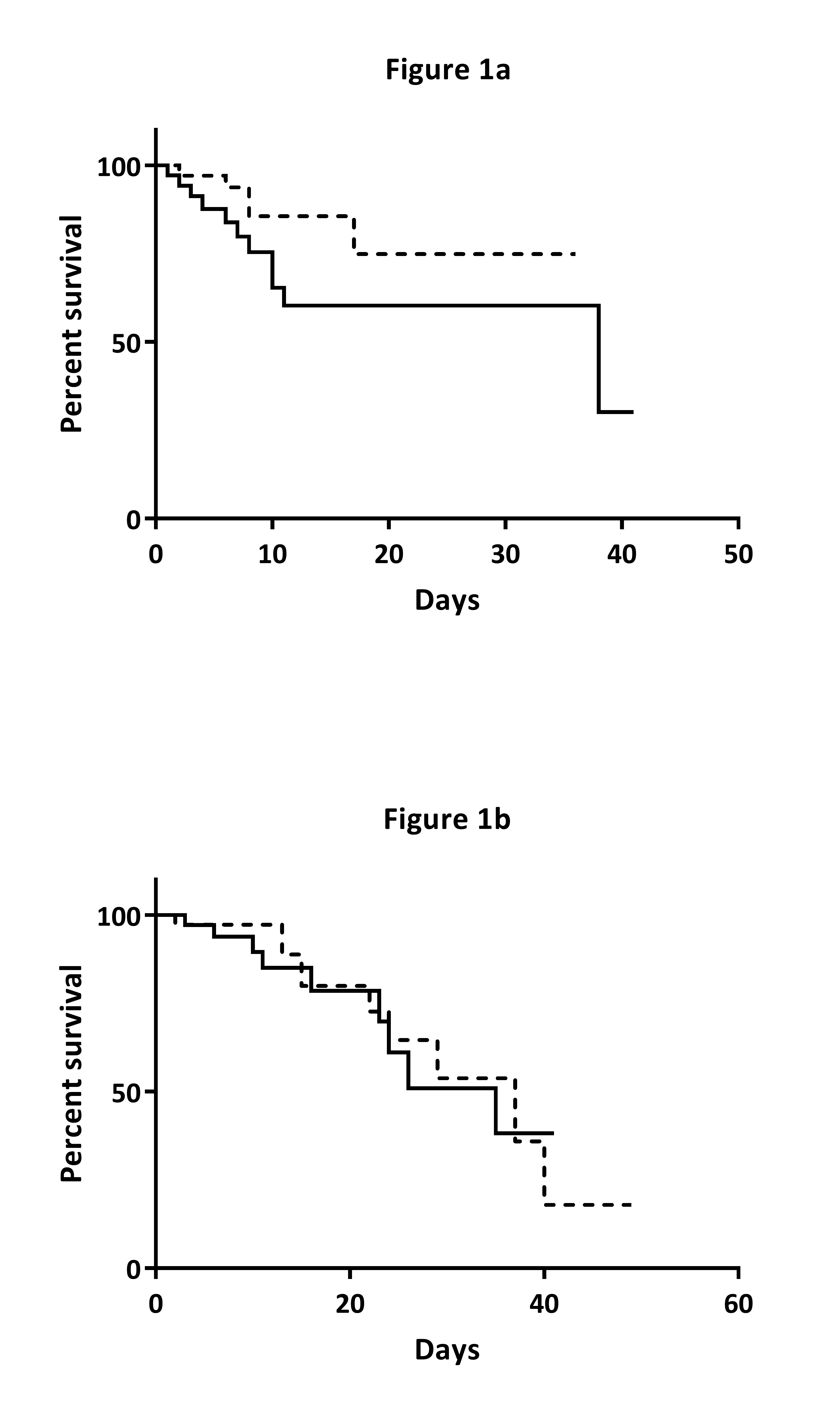

Supplement: Supplementary file 1 [file hygsup.zip › S0950268822000267sup001.tif]
